# Feasibility pilot of a novel coaching intervention to optimize cannabis use for chronic pain management among Veterans

**DOI:** 10.1186/s42238-025-00265-z

**Published:** 2025-01-25

**Authors:** Kevin F. Boehnke, Gabrielle Bowyer, Jenna McAfee, Tristin Smith, Catherine Klida, Vivian Kurtz, Evangelos Litinas, Poonam Purohit, Anne Arewasikporn, Dana Horowitz, Laura Thomas, Jennifer Eckersley, Mia Railing, David A. Williams, Daniel J. Clauw, Kelley M. Kidwell, Amy S. B. Bohnert, Rachel S. Bergmans

**Affiliations:** 1 Anesthesiology Department, University of Michigan Medical School, Ann Arbor, MI 48105 USA; 2 Chronic Pain and Fatigue Research Center, University of Michigan Medical School, Ann Arbor, MI USA; 3 Michigan Psychedelic Center, University of Michigan, Ann Arbor, MI USA; 4 Green Pillar Consulting, Ann Arbor, MI USA; 5 Department of Biostatistics, School of Public Health, University of Michigan, Ann Arbor, MI USA; 6 Veterans Affairs Ann Arbor Healthcare System, Ann Arbor, MI USA

**Keywords:** Cannabis, Coaching, Feasibility, Clinical trial, Cannabidiol, THC

## Abstract

**Introduction:**

Chronic pain is common among Veterans, some of whom use cannabis for pain. We conducted a feasibility pilot study of a novel coaching intervention to help Veterans optimize use of medical cannabis products for pain management (NCT06320470).

**Methods:**

The intervention drew from scientific literature, consultation with cannabis experts, Veteran input via a Community Advisory Board, and tenets of motivational interviewing. Participants were Veterans with chronic pain who endorsed current use or interest in using cannabis for pain management. Participants received up to 4 individual coaching sessions via videoconference, spaced approximately 2 weeks apart. We assessed feasibility (adherence, satisfaction, acceptability) and preliminary effects on pain symptoms 14 weeks after baseline. The primary outcome was the Patient Global Impression of Change (PGIC), and exploratory outcomes included domains from the Patient-Reported Outcomes Measurement Information System (PROMIS)-29.

**Results:**

Of 22 enrolled participants, 17 attended 4 coaching sessions, 2 attended 3 sessions, and 2 attended 2 sessions. Among those who completed end of intervention surveys (16/21), 87.5% were very or completely satisfied with the intervention and 81.3% rated coaching as very or extremely helpful. All participants reported improvement on the PGIC, with 63% reporting much or very much improvement. Participants reported statistically significant decreased pain intensity (7.1/10 vs. 5.7/10) and pain interference (T-score 66.3 vs. 61.8), and increased social satisfaction (T-score 41.4 vs. 44.3). Participants noted helpful intervention factors, including co-developing a personalized plan, discussing questions/concerns, and trying different approaches to cannabis-based treatment.

**Conclusions:**

In this feasibility pilot study of coaching on cannabis use for chronic pain among Veterans, participants were satisfied with the intervention and reported clinically significant improvements in pain symptoms. Our results support evaluating this intervention in a larger, efficacy trial.

## Introduction

An estimated 20.4% of adults in the United States report chronic pain, defined as pain lasting 3 or more months (Zelaya et al, 2020). Chronic pain is even more common among Veterans, affecting up to 30% in the general Veteran populace, who sustain service-related injuries and are exposed to a wide range of physiological and psychological stressors (Qureshi 2023). The financial burden of chronic pain has been estimated at $560 billion annually in direct medical costs, lost productivity, and disability-related expenses (Institute 2011). Unfortunately, pharmacological treatments for chronic pain tend to offer partial relief in only a subset of patients, which are often discontinued due to aversive side effects and limited pain relief (Chou et al. 2017; Finnerup et al. 2015; Hauser et al. 2010; Wolfe et al. 2013). For example, use of opioids for chronic pain is common despite there being little evidence supporting their benefits and much evidence supporting potential harm (Dowell et al. 2016).

Many Veterans have sought and advocated for alternatives for pain management, including cannabis and its constituent cannabinoids (Veterans 2024; Jaeger 2024), which include delta-9-tetrahydrocannabinol (THC) and cannabidiol (CBD). Indeed, data from the nationally representative National Health and Resilience in Veterans Study estimated that 12% of US Veterans report cannabis use in the past 6 months, with 1.5% obtaining a medical cannabis card (Hill 2019). Further, data from the National Epidemiologic Survey on Alcohol and Related Conditions-III showed that the prevalence of cannabis use is higher among Veterans with pain than those without (9% vs. 6.2%) (Enkema et al. 2022). Due to an increasingly tolerant legal landscape, cannabis is now legal for medical use in 38 states (Boehnke et al. 2022). The most common reason people obtain medical cannabis licenses is for chronic pain (Boehnke et al. 2022; Boehnke et al. 2019; Boehnke 2024), with many individuals reporting substantial pain relief and use of cannabis as a substitute for traditional pain medications (Boehnke et al. 2016; Boehnke et al. 2019; Piper et al. 2017; Corroon et al. 2017). Yet clinical trial results on cannabis’s analgesic effects remain inadequate due to historical legal restrictions on research and inconsistencies across studies (Stith 2016; Fisher et al. 2021), Indeed, one recent commentary termed the clinical trial literature a “methodological minefield” due to variable dosing paradigms, routes of administration, cannabinoids used, and lack of long-term studies (Hauser et al. 2018). As such, there is much uncertainty among treatment providers about how to counsel patients on effective use of cannabinoid-based therapies.

Recently, an interdisciplinary team of researchers and healthcare professionals from Canada proposed clinical practice guidelines regarding cannabis for the management of chronic pain based on a systematic review of existing literature (Bell 2023). However, studies that operationalize these guidelines have not been empirically tested (both generally and among Veterans), nor are there any clinical trials to our knowledge that assess implementation of these guidelines using commercial cannabis products. Further, conversations about cannabinoid-based therapies for pain remain largely removed from U.S. clinical practice, and many Veterans use cannabis products of various routes of administration (e.g., smoking, eating, vaporizing, oils) and cannabinoid content (e.g., cannabidiol [CBD], delta-9-tetrahydrocannabinol [THC]) often without direct guidance or support from an informed provider (Hill 2019; Bergmans 2024). Use patterns often diverge from the available evidence and what guidance from modified Delphi panel studies of cannabis experts suggests to be safest and most effective (Boehnke et al. 2019; Boehnke et al. 2022; Bhaskar et al. 2021; MacCallum et al. 2021; MacCallum and Russo 2017), potentially inviting unwanted side effects that may interfere with possible benefits.

As such, we developed and conducted a feasibility pilot study of a coaching intervention drawn from the scientific literature, the clinical expertise of scientists and physicians who work directly with those using medical cannabis, and motivational interviewing. We chose motivational interviewing as the counseling style for our intervention as it is an evidence-based, person-centered approached that has been tested in other substance use contexts (Bohnert et al. 2016; Blow et al. 2017). In this intervention, participants engaged with trained cannabis coaches (research interventionists with a Master of Social Work degree) to learn how to methodically use self-selected cannabis products for pain management and received personalized, scientifically guided content related to their symptoms. In this manuscript, we outline the intervention development and results from our study, which assessed participant adherence and satisfaction with the intervention, as well as explored preliminary effects on chronic pain symptoms. Our primary hypothesis was that this intervention would be feasible and well received, with participants reporting satisfaction with the intervention and improved patient-reported global symptoms.

## Methods

### Study participants and recruitment

We aimed to enroll at least 20 participants to assess feasibility, participant satisfaction, and potential challenges of delivering this novel intervention, as well as preliminary data on outcomes of the intervention on pain and related symptoms. We recruited from August 7–29, 2023 by first contacting Veterans who participated in other studies related to cannabis use and chronic pain, including a qualitative study (Bergmans 2024) and a longitudinal registry called MIVetsCan. We also recruited participants by posting on the University of Michigan’s research website (UMHealthResearch.org). The last study assessment was completed on December 5, 2023.

Participants were adults aged 18 or older who were Armed Services Veterans with self-reported chronic pain lasting 3 or more months and were interested in using or currently using cannabis for chronic pain management.

Participants completed informed consent documents electronically using the MyDataHelps app (CareEvolution, Ann Arbor, MI), which provides a secure platform for collecting protected health information remotely and has been used in large well-known studies such as the NIH All of Us Study and the eFramingham study (Quer et al. 2021; Pathiravasan et al. 2021; Lin et al. 2020; McManus et al. 2019). In addition to informed consent, the MyDataHelps app served as the vehicle for data collection via study questionnaires and synced activity tracking devices (i.e., Fitbits) as well as automated study notifications. The app also displayed a digital dashboard so that participants could track changes in their pain-related symptoms and cannabis use over time.

The University of Michigan Institutional Review Board (IRB) approved all study methods and procedures under HUM00231159. Participants received up to $200 for participating in the study, with payment disbursed after each completed survey. This trial was registered to clinicaltrials.gov as NCT06320470.

### Intervention development

We developed the educational intervention with insight from multiple sources, including the most recent and relevant scientific literature (Bell 2023; Bhaskar 2021; MacCallum 2018; National Academies of Sciences, Engineering, and Medicine 2017; Boehnke et al. 2024), consultation from a medical cannabis expert who had substantial clinical experience working with medical cannabis patients (co-author EL), input from Veterans via a Community Advisory Board (CAB), and informed by the tenets of Motivational Interviewing (MI). We developed a manual for this intervention, drawing from several comprehensive reviews on the effects of cannabinoids and how they related to chronic pain symptoms (Bell 2023; Bhaskar 2021; MacCallum 2018; National Academies of Sciences E, and Medicine 2017). Two Master’s-level therapists (GB, DH) delivered the intervention to study participants. Required hiring criteria for this role included past training and experience with client therapy, manualized intervention delivery, and MI. The last criterion was derived from the study team’s history with conducting motivational interviewing-based interventions in previous studies (Bohnert et al. 2016; Blow et al. 2017).

To center Veterans’ perspectives, we used a community-engaged approach throughout the study design process. These efforts included soliciting feedback from the Ann Arbor Veteran Affairs Veterans Research Engagement Council (VREC) and establishing a Community Advisory Board (CAB) to help shape research priorities, including providing input on study design and educational materials. The CAB is primarily composed of Veterans who have chronic pain, representatives of organizations that provide services to Veterans, as well as medical providers who work with Veterans. The CAB influenced the development of the intervention by helping us to ensure its relevance to Veteran communities and verifying that the design was responsive to Veteran priorities and perspectives. For example, we modified the coaching intervention to add extra information related to cannabis product safety, dosing, and cannabis legality based on the CAB’s feedback. We also sought feedback and approval for our recruitment materials and approach with the CAB and VREC, who suggested additional opportunities and settings for engaging with Veterans such as Veteran resource fairs.

We designed a manual for the coaching intervention that draws from the cannabis literature as well as the spirit and guidelines of MI. MI is an evidence-based, person-centered approach that helps to spur behavioral change through opportunistic conversing rather than prescribed or directed recommendations (Miller 2012). MI encourages a curious, non-hierarchical, and non-judgmental conversational style in which the coach supports and affirms participant autonomy. The delivery of a MI-based coaching intervention is fundamentally relational: coaches demonstrate partnership, convey acceptance, cultivate compassion, and emphasize empowerment. In addition to borrowing from the humanistic philosophies of MI, we directly incorporated MI-specific skills and tasks into the intervention: open questions, affirmations, reflections, and summaries. Coaches use each as conversational vehicles to favor participant language for change versus the status quo. Open-ended questions are featured prominently in the didactic portions of the manual, such as the “elicit-provide-elicit” structure to first explore what the participant already knows, then requesting permission to share relevant interviewer knowledge, and finally exploring the participant's response. Coaches then selected salient elements from participant speech to reflect back as a demonstration of active listening. More complex reflections include sharing coach observations about participant’s nonverbal cues or hypotheses about what the client may be feeling or thinking but have not explicitly shared. Reflections impart a sense of empathy and build rapport. The spirit and tasks of MI informed the style in which the standardized manual was delivered to participants; coaches identified relevant MI skills to use within each section to ensure participant engagement, partnership, and empowerment. The manual was unscripted to allow the coaches therapeutic creativity on connecting the content to the individual participant and their lived experiences (e.g., cannabis naïve, their specific pain symptoms, etc.).

Our goal was to empower participants with sound scientific information via encouraging partnerships to achieve confidence in pain symptom self-management with self-selected medical cannabis products. Participants met with the same coach (GB or DH) throughout the trial to enable relationship-building with ample validation and support in the process of change. Further, the coaching intervention was consistently adaptive to participant feedback. During each, we asked participants to rate their confidence in using cannabis for pain management on a 0–10 scale (0 = not at all confident, 10 = completely confident) Likert scale. Coaches then explored what made the participant choose their rating, why the rating was not lower (e.g., a 6 rather than a 4), and whether the participant could identify factors that might increase their confidence in the future. Discrepancies between the selected number and other statements made by participants were also explored, such as a low number coupled with a demonstrated high degree of competence or vice versa. With safety, accuracy, and empowerment in mind, coaches identified and addressed misinformation or discouragement.

### Intervention description and delivery

**Fig. 1:**
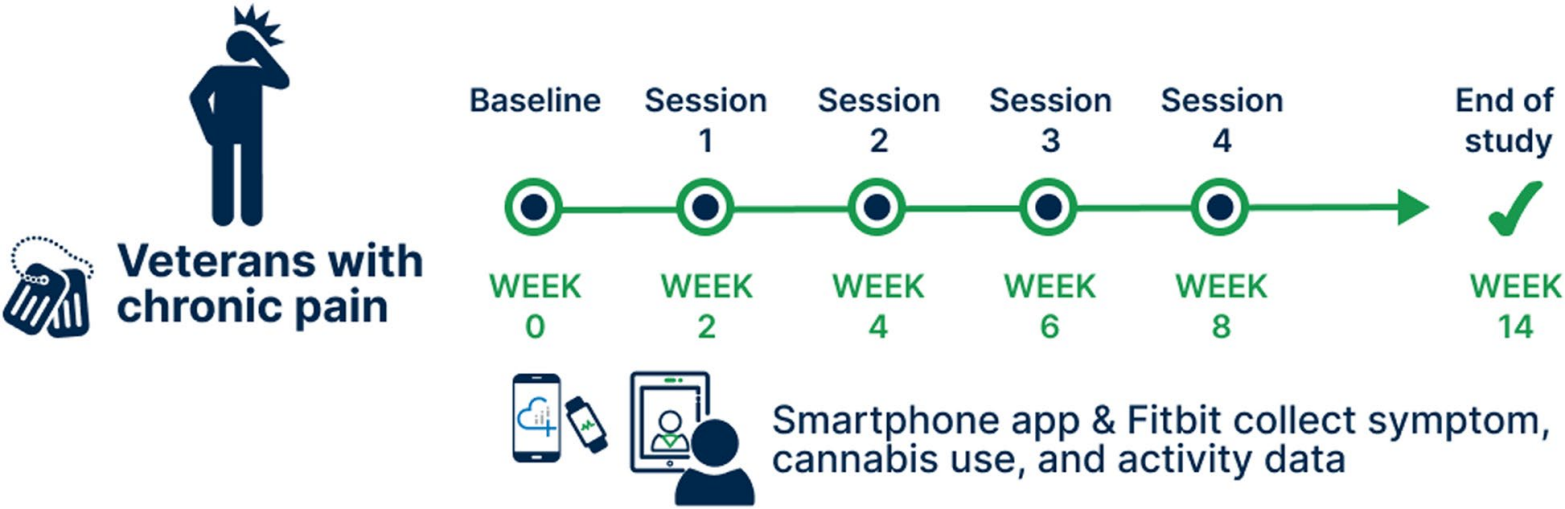
Study design

Figure 1 provides an overview of the study design. In brief, this 14-week long intervention encompassed 4 cannabis coaching visits and access to educational materials about cannabis. After enrollment, we gave study participants access to the Cannabis Guide, which is a set of infographics delineating steps for using cannabis products to achieve pain management goals aligned with expert guidance published in the scientific literature (Bell 2023; Bhaskar 2021; MacCallum 2018; Boehnke et al. 2024). The Cannabis Guide briefly summarizes key didactic elements from the manual. We then assigned participants to a cannabis coach who offered up to 4 virtual coaching sessions scheduled approximately 2 weeks apart. We followed participants for 6 weeks after the final coaching session, with the primary endpoint assessments occurring at 14 weeks after baseline.

#### Coaching session content (overviewed in Table 1)

**Table 1:** Overview of manualized intervention

Coaching domains and content	Description
Anonymity and confidentiality	Note that sessions are recorded and that everything will be anonymous and confidential
Goal setting	Elicit health goals related to pain as well as how participant hopes cannabis might help achieve those goals
Pain symptoms	Coach elicits information related to pain, sleep, anxiety, and any other symptoms of interest
Cannabis use history	Coach history of past and current use, including intentions (medical vs. non-medical), frequency of use, methods of use, dosing, cannabinoid content, and noted effects on symptoms
Clinical pearls (Bell 2023; Bhaskar 2021; MacCallum 2018; Boehnke et al. 2024)	
* Routes of administration*	Education on routes of administration, including effect onset and how to fit use patterns together
* Cannabinoid content (CBD, THC)*	Description of CBD effects (e.g., non-intoxicating, anxiolytic, potentially anti-inflammatory) vs. THC effects (e.g., gets you ‘high’, sleep effects)
* Dosing and titration*	Suggest starting dose aligned with what was suggested in modified Delphi panels and emphasis on “Start low, go slow” titration
* Use timing*	Aligning use timing with symptoms and practical considerations, such as not being high while operating a vehicle or at work
* Storage*	Keep in dark cool places. Keep edibles carefully hidden especially if children or pets present in household
* Side effects*	Overview of common effects of cannabis, especially related to plan for next time and current use patterns
Development of plan for future use	Co-developed and dependent on participant preferences. May include identification and discussion of barriers and facilitators, plan for use as well as practical information like how to navigate a dispensary visit
Confidence ruler	0–10 assessment of confidence, followed by discussion of why the confidence is not higher or lower
Plan for next time	Summary of what was discussed and how participant is planning to proceed in the interim. Followed up by email with key learning points and summary of plan for next time

Sessions drew from the tenants of Motivational Interviewing for the purpose of content delivery

The first coaching session was on average 57.8 min (SD = 9.9 min) and resembled an “intake” visit. Coaches asked participants to provide detailed information on their cannabis use history, pain experience and symptoms, as well as co-occurring issues such as anxiety and sleep problems. Accurate histories enabled coaches to tailor cannabis plans to each participant’s set of symptoms and previous helpful or unhelpful experiences with cannabis therapeutics. The coaches also asked participants to share relevant health-related goals that they hoped to achieve through cannabis-based treatment. These goals served as useful signposts for coaches to revisit over the course of the intervention, and included stress management, sleeping well, better pain control, reduction in side effects, going back to school, and better relationships with others.

**Fig. 2:**
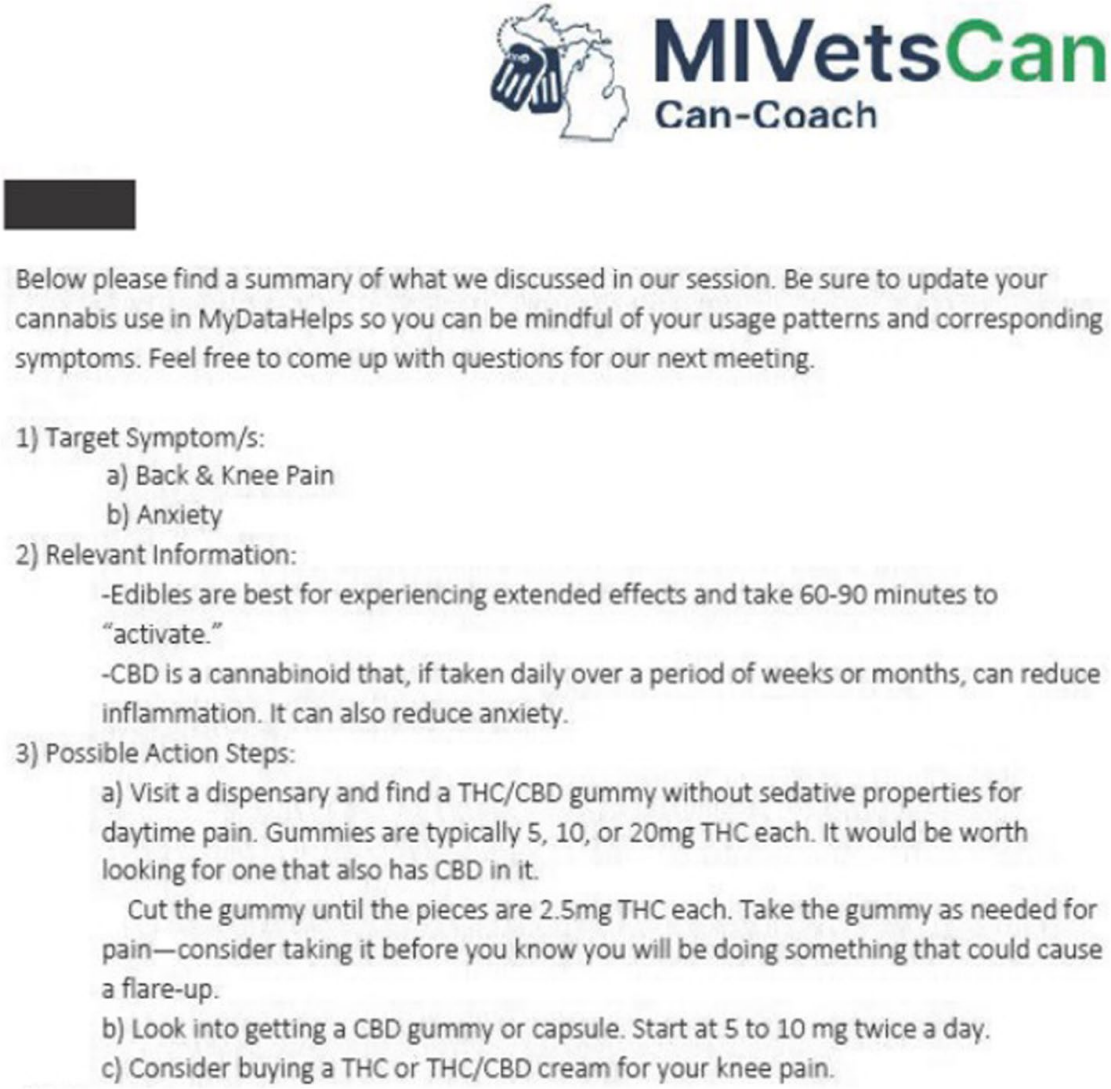
Example post-visit summary email from coach to a study participant who reported daily back and knee pain, current use of smoked cannabis (THC), and desire to experiment with alternate routes of administration

Once coaches collected histories, noted goals, and began to develop rapport with participants, they moved onto the didactic portion of the first session: the domains of cannabis use. Referencing the Cannabis Guide, coaches used the motivational interviewing task of “elicit-provide-elicit” with the following topics to partner with participants increase their understanding of cannabis products: differential effects of THC and CBD, routes of administration available (i.e. smoking, vaporizing, eating, topicals, tinctures) and how their effect onsets and durations differ, methodical “start low, go slow” dosing strategy, effective timing of use (aligning therapeutic effect with onset of symptoms), safety considerations, and side effects. This task allowed coaches to learn what all participants, regardless of their history of cannabis use, already knew about each clinical pearl before providing information to fill in any gaps or correct misinformation. This also helped coaches to optimize their time with participants by reducing redundant didactics. The coaches then partnered with the participants to come up with a realistic cannabis use plan given the participant’s pain symptoms, their history of cannabis use, their health goals, and what was realistic to integrate into their lives. The coaches encouraged tracking symptoms and cannabis use via MyDataHelps so that this information could inform participant choices about current and future cannabis use. The first session concluded with coaches collaborating with participants to decide the appropriate first steps in optimizing their use (Fig. 2).

The three following sessions served to reinforce content from the first session, address any concerns that arose since the last session, and discuss potential changes to the self-directed treatment approach (mean duration = 30.3 min; SD = 7.8 min). Coaches encouraged participants to share any behavioral changes related to cannabis use, the status of their pain symptoms, and side effects. In the spirit of patient-centered care, coaches provided individualized guidance reflective of each participant’s goals, needs, and limitations. For instance, some participants wanted to experiment with CBD products only. Some did not want to smoke or vaporize, but rather focus on ingestion, tinctures, or topicals. Others felt the most relevant goal was to address insomnia, which exacerbated their pain symptoms during the day. The final session mimicked other follow-up sessions, but additionally included dialogue about how participants could independently adjust and monitor their use routines post intervention, identifying supports and lessons learned.

## Measures

### Demographics and cannabis use

We assessed participant sex at birth, age, employment status, and past cannabis use. To measure cannabis use we assessed any use in the past, as well as timing of past use (1–3 months previously, 3–6 months previously, > 6 months previously, never).

### Feasibility outcomes

We assessed participant adherence to the protocol and retention in the study. We also assessed participant satisfaction with the intervention using a 5-point Likert scale ranging from *not at all satisfied* to *extremely satisfied*. Participants indicated whether the number of coaching sessions was too many, too few, or the right amount. They also rated the length of coaching sessions and time between coaching sessions (i.e., two weeks) as too long, about right, or too short. Additionally, we assessed perceptions of helpfulness of coaching sessions using a 5-point Likert scale ranging from *not at all satisfied* to *extremely satisfied*. Lastly, we asked participants several open-ended questions about the most and least helpful aspects of the intervention and solicited feedback for overall program improvement.

### Fidelity: adherence to content and motivational interviewing tenets

To ensure fidelity to the treatment manual, both interventionists conducted mock sessions with volunteers and received feedback from a clinical supervisor before delivering study sessions. We recorded study sessions and randomly selected 20% of sessions for coding with the Motivational Interviewing Treatment Integrity (MITI), a coding system that gauges how well the interviewer is adhering to Motivational Interviewing (Moyers 2014). The MITI has two components: the global assessment dimensions and the behavior counts. For this analysis of a MI-based intervention, we assessed only the global scores.

Using a random 20-min segment of each randomly selected session, trained MITI coders listened and assigned a single number global score on a five-point scale based on an overall impression. The four global assessment dimensions are divided into two technical skills and two relational skills. The technical skills are cultivating change talk and softening sustain talk (fair score = 3, good = 4). The relational skills are using partnership and displaying empathy (fair score = 3.5, good = 4). Finally, the same 20% of sessions were coded by trained staff for adherence to 10 separate categories of content, such as goal setting, ascertaining cannabis use history, assessing pain symptoms, discussing safety and side effects. For fidelity checks, we considered 80% or higher content adherence to be passing.

### Preliminary effectiveness outcomes

#### Primary outcome

Our primary preliminary effectiveness outcome measure was the Patient Global Impression of Change (PGIC) (Farrar et al. 2001), a 1-item measure that assesses patient perceptions of intervention success and is a well-established indicator of whether patients experience meaningful improvement from a given treatment. This 7-point Likert scale ranges from*very much worse* to *very much improved*.

#### Exploratory outcomes: domains relevant to chronic pain

Participants completed the Patient-Reported Outcomes Measurement Information System (PROMIS) 29 + 2 v2.1, which measures physical function, pain interference, pain intensity, anxiety, fatigue, cognitive function, social satisfaction, and sleep disturbance through various Likert scales (Cella et al. 2011; Hays 2018). These items generate raw scores, which are then converted to a standardized T-score for each subscale using the HealthMeasures Scoring Service. These scales range from 0–100 (mean = 50, standard deviation = 10).

Participants also completed the Positive and Negative Suicide Ideation Inventory (PANSI) (Muehlenkamp et al. 2005). The PANSI is separated into the 8-item negative suicide ideation subscale that assesses suicidal vulnerability and the 6-item positive ideation subscale that assesses protective factors. To account for missing responses to some questions, average scores were calculated for both the subscales if fewer than 3 questions were missing.

### Statistical analysis

We characterized participant demographics, self-reported cannabis use frequency, overall satisfaction with the intervention, and PGIC scores using descriptive statistics using all data from those who completed assessments at each timepoint. Among participants who completed the final assessments, we assessed differences between baseline and post-intervention PROMIS and PANSI subscale scores using paired t-tests. We used R (version 4.1.1) for all analyses.

## Results

### Demographics and safety

**Fig. 3:**
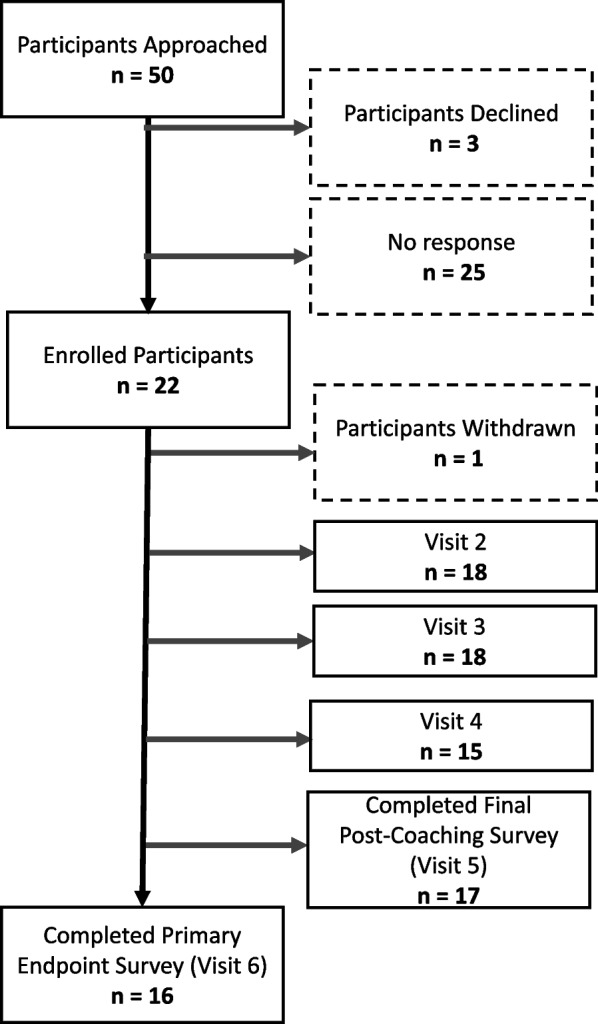
CONSORT diagram of recruitment

We approached 50 potential participants and enrolled a total of *n* = 22 (Fig. 3). One withdrew immediately after consent (did not complete baseline assessments) because they had a family matter emerge and could not commit adequate time to the study. Of the 21 remaining participants, 76.2% were male, 81% were White, 47.6% were disabled or unable to work, and 38.1% were retired (Table 2). Over three quarters (76.2%) had prior experience with cannabis while 23.8% did not. Overall, 17 participants attended all 4 coaching sessions, 2 attended 3 coaching sessions, 2 attended 2 coaching sessions. At the end of intervention (week 8), 17/21 (81%) completed survey assessments and 16/21 (76.2%) completed survey assessments at the final study visit (week 14). Among the 5 participants who did not complete the final survey assessment, one indicated they did not complete the survey due to being too busy, and the other four did not give an explanation for why they did not complete the surveys. There were no adverse events (AEs) related to the study, and one study participant experienced three unrelated adverse events, which included hospitalization and significant disability due to malaria.

**Table 2:** Participant demographics

Characteristic	Participants who completed baseline assessments (*n* = 21)	Participants who completed the final study assessments (*n* = 16)	Participants who did not complete the final study assessments (*n* = 5)
**Age Group**			
Mean (SD)	57.7 (12.7)	56.4 (12.1)	61.8 (15.1)
30–34	1 (4.8%)	1 (6.3%)	0 (0.0%)
40–44	2 (9.5%)	1 (6.3%)	1 (20.0%)
45–49	2 (9.5%)	2 (12.5%)	0 (0.0%)
50–54	4 (19%)	3 (18.8%)	1 (20.0%)
55–59	1 (4.8%)	1 (6.3%)	0 (0.0%)
60–64	6 (28.6%)	5 (31.3%)	1 (20.0%)
65–69	1 (4.8%)	1 (6.3%)	0 (0.0%)
75–79	4 (19%)	2 (12.5%)	2 (40.0%)
**Sex at Birth**			
Male	16 (76.2%)	12 (75.0%)	4 (80.0%)
Female	5 (23.8%)	4 (25.0%)	1 (20.0%)
**Race**			
Black or African American	2 (9.5%)	2 (12.5%)	0 (0.0%)
White/Caucasian	17 (81%)	12 (75.0%)	5 (100.0%)
Two or More Races Reported	1 (4.8%)	1 (6.3%)	0 (0.0%)
Other	0 (0%)	0 (0%)	0 (0.0%)
Refused to Answer	1 (4.8%)	1 (6.3%)	0 (0.0%)
**Hispanic Origin**			
No	20 (95.2%)	15 (93.8%)	5 (100.0%)
Yes	0 (0%)	0 (0%)	0 (0.0%)
Refused to Answer	1 (4.8%)	1 (6.3%)	0 (0.0%)
**Employment Status**			
Self-Employed	1 (4.8%)	0 (0%)	1 (20.0%)
A homemaker	1 (4.8%)	1 (6.3%)	0 (0.0%)
A student	1 (4.8%)	1 (6.3%)	0 (0.0%)
Retired	8 (38.1%)	7 (43.8%)	1 (20.0%)
Unable to work/disabled	10 (47.6%)	7 (43.8%)	3 (60.0%)
**Last Use of Cannabis**			
Never (cannabis naïve)	5 (23.8%)	4 (25.0%)	1 (20.0%)
> 6 months ago	4 (19.0%)	4 (25.0%)	0 (0.0%)
3–6 months ago	1 (4.8%)	1 (6.3%)	0 (0.0%)
1–3 months ago	11 (52.4%)	7 (43.8%)	4 (80.0%)

Continuous age reported as mean (SD), all other values reported as n (%). In total, 21 participants completed the baseline survey. Of these participants 16 completed the final follow-up questionnaire

### Intervention delivery

An average of 94% of the intended intervention content was delivered across all sessions. One session fell below the 80% content adherence threshold (77%). The average technical and relational global scores for study sessions were 4.74 and 4.82 respectively, demonstrating strong adherence to the manual. The two coaches had sufficient time and bandwidth to deliver the intervention to study participants.

### Feasibility outcomes

Among those who completed the intervention (*n* = 16), 87.5% were very or completely satisfied with the intervention and 81.3% rated coaching as very or extremely helpful (Table 3). Similarly, 87.5% thought that the length of coaching sessions was “about right” and 75% thought that the 2-week length between coaching sessions was “about right.” At week 14, 50% thought that the number of coaching sessions was about right, a decrease from the 76.5% (13/17) participants who thought that the number of coaching sessions was about right just after the final coaching session (week 8). Qualitatively, participants reported positive views of the intervention, noting the helpfulness of developing a personalized plan, connecting with the coach, ability to address concerns, and trying different strategies. In open-ended feedback, some participants expressed frustration about not being given specific brand recommendations and some reported wanting more coaching sessions. This feedback was especially emphasized among those who had no prior experience with cannabis.

**Table 3:** Participant satisfaction

	**Final coaching session: Week 8 (*n* = 17)**	**Primary endpoint: Week 14** **(*n* = 16)**
**Satisfaction with Cannabis Coaching Session Today**		
Not at all satisfied	0 (0%)	-
Somewhat satisfied	0 (0%)	-
Satisfied	3 (17.6%)	-
Very satisfied	2 (11.8%)	-
Completely satisfied	12 (70.6%)	-
**Satisfaction with Cannabis Coaching Session Overall**		
Not at all satisfied	0 (0%)	0 (0%)
Somewhat satisfied	0 (0%)	0 (0%)
Satisfied	0 (0%)	2 (12.5%)
Very satisfied	4 (23.5%)	6 (37.5%)
Completely satisfied	13 (76.5%)	8 (50.0%)
**Number of Cannabis Coaching Sessions**		
Too many	0 (0%)	0 (0%)
About right	13 (76.5%)	8 (50.0%)
Not enough	4 (23.5%)	8 (50.0%)
**Length of Each Cannabis Coaching Session**		
Too long	0 (0%)	0 (0%)
About right	16 (94.1%)	14 (87.5%)
Too short	1 (5.9%)	2 (12.5%)
**Length Between Each Cannabis Coaching Session (2 weeks)**		
Too long	0 (0%)	0 (0%)
About right	15 (88.2%)	12 (75.0%)
Too short	2 (11.8%)	4 (25.0%)
**Helpfulness of Cannabis Suggestions in Coaching Sessions**		
Not at all helpful	0 (0%)	0 (0%)
Somewhat helpful	1 (5.9%)	1 (6.3%)
Moderately helpful	0 (0%)	2 (12.5%)
Very helpful	6 (35.3%)	8 (50.0%)
Extremely helpful	10 (58.8%)	5 (31.3%)

14 participants answered questions at both visit 5 and visit 6. 19 of the 21 participants answered feedback questions at either visit 5 or 6 (or both)

### Clinical outcomes

Overall, 63% of participants reported much or very much improvement on the PGIC (Table 4). Relative to baseline, participants reported statistically significantly decreased pain intensity (7.1/10 vs. 5.8/10; *p* = 0.01), pain interference (PROMIS T-score 66.5 vs. 62.0; *p* < 0.01), and increases in social satisfaction (PROMIS T-score 41.4 vs. 44.3, *p* = 0.01). No other changes in symptoms were statistically significant (Table 5).

**Table 4:** Participant-Reported Patient Global Impression of Change (PGIC)

Patient Global Impression of Change	Participants who Completed Final Survey (*n* = 16)
Very Much Improved	1 (6.3%)
Much Improved	9 (56.3%)
Minimally Improved	6 (37.5%)
No Change	0 (0%)
Minimally Worse	0 (0%)
Much Worse	0 (0%)
Very Much Worse	0 (0%)

Participant-reported Patient Global Impression of Change, responses reported as n (%). “Much improved” and “Very much improved” are considered clinically significant

**Table 5:** Changes in PROMIS and PANSI Scores from Baseline among the *n* = 16 participants who completed the final survey

Measure	Baseline	Visit 6 Follow-Up	Change
PROMIS Physical Function	37.71 (5.35)	38.01 (5.35)	0.29 (2.05); 16
PROMIS Cognitive Function	46.01 (4.68)	47.86 (6.36)	1.85 (7.05); 16
PROMIS Anxiety	59.01 (7.07)	58.27 (6.41)	−0.74 (5.85); 16
PROMIS Depression	54.87 (10.18)	55.41 (10.23)	0.55 (6.13); 15
PROMIS Fatigue	61.06 (5.58)	59.15 (7.67)	−1.91 (5.67); 16
PROMIS Sleep Disturbances	55.49 (3.74)	56.43 (2.28)	0.93 (3.73); 16
PROMIS Social Satisfaction	41.45 (8.75)	44.33 (10.92)	2.89 (3.90)*; 15
PROMIS Pain Interference	66.49 (7.33)	62.01 (8.31)	−4.49 (4.84)*; 15
PROMIS Pain Intensity	7.06 (1.18)	5.81 (1.64)	−1.25 (1.81)*; 16
PANSI Positive Ideation	3.27 (0.90)	3.25 (0.97)	−0.02 (0.79); 15
PANSI Negative Ideation	1.32 (0.54)	1.39 (0.65)	0.07 (0.32); 14

Summary statistics for PROMIS and PANSI measures reported as mean change (SD); n. All values are t-scores with exception to pain intensity which ranges from 0–10. Higher physical function, cognitive function, and social satisfaction scores and lower anxiety, depression, fatigue, sleep disturbances, pain interference, and pain intensity scores are indicative of better overall functioning. Higher scores on the negative suicide ideation and lower scores on the positive ideation are indicative of greater risk for suicidal behavior. *Denotes *p*-values < 0.05 using paired t-tests

## Discussion

In this feasibility pilot study, we demonstrated that a novel cannabis coaching intervention focused on improving chronic pain symptoms among Veterans was feasible and acceptable for study participants and was associated with improvements in pain symptoms. Generally, participants were adherent, satisfied, and felt that the intervention was helpful. These perceptions were confirmed by clinical outcomes as all participants reported global symptom improvement per the PGIC, with 63% reporting much or very much improvement, which are thought to be clinically significant changes in previous chronic pain research (Farrar et al. 2001). Further, participants reported improved pain and pain interference, with the effect size for improvements in pain interference exceeding those that others have considered to be clinically significant for people with chronic pain: 4.5 points in our study vs. 2–3.5 points as a minimally important difference (Chen et al. 2018). While several recent articles have made suggestions about how to use cannabis in the chronic pain management context including a proposed clinical guideline and modified Delphi studies (Bell 2023; Bhaskar 2021; MacCallum 2018; National Academies of Sciences E, and Medicine 2017), to our knowledge this study is the first to empirically test whether coaching informed by evidence-based guidelines affects chronic pain outcomes. Importantly, our intervention provided guidance and education that allowed participants to select and use their own cannabis products from the existing legal marketplace, empowering them to make decisions that addressed their individual needs and symptoms.

This study builds upon the existing pragmatic trial literature of medical cannabis in the U.S. For example, a recent pragmatic clinical trial compared the effects of obtaining a medical cannabis license vs. a waitlist control among people with pain, insomnia symptoms, and anxiety or depressive symptoms (Gilman 2022). This trial showed that immediate treatment led to improvements in sleep but no meaningful improvements in pain, anxiety, or depression. Further, immediate treatment was associated with higher cannabis use disorder symptoms. These results suggest that simply providing access to cannabis may have some benefits but also some risks. These risks are likely heightened by the current marketplace emphasis on products with high quantities of THC (Pennypacker 2022)and a dispensary culture in which many dispensary employees receive mixed medical education, often relying on their own personal experiences or those of their coworkers for making recommendations about medical use (Haug et al. 2016; Merlin 2021). Further, many have expressed concern about disclosing cannabis use to their primary care providers due to legal concerns, stigma, and perceptions that their care providers do not have adequate knowledge about cannabis or the ability to adequately integrate it into treatment (Bottorff et al. 2013; Lau et al. 2015; Lau et al. 2015; Boehnke et al. 2021; Holman et al. 2022).

Of note, our results align with a substantial body of real-world evidence showing that people obtain substantial pain management benefits when using cannabis products with guidance from trained clinical professionals. For example, in Israel (a country with a federal medical cannabis program), researchers conducted a prospective cohort study of 367 individuals with fibromyalgia, who were offered a gradual titration approach under the guidance of a certified nurse who provided advice on strains and routes of administration (Sagy 2019). Participants reported significantly reduced pain intensity (median 9/10 at baseline to 5/10 at 6 month follow-up) and improved quality of life, with reportedly mild side effects. Similarly, a prospective cohort of 2,736 elderly individuals with mixed conditions, including chronic pain showed substantial improvements after 6 months of cannabis therapy that was guided by a nurse who made recommendations related to cannabis varieties and routes of administration (Abuhasira et al. 2018). As with Sagy et al., participants generally reported minor adverse events, with the most common being dizziness and dry mouth. These results emphasize how clinical guidance can help reduce side effects while maximizing treatment benefit. As with any pain medication, cannabis will not provide relief for all individuals (Nutt 2020), but evidence thus far suggests that skilled guidance enhances the potential for pain management.

### Implications

Because cannabis remains federally illegal and there are no cannabinoid products approved for pain by the U.S. Food and Drug Administration (Boehnke et al. 2024), people using cannabis for pain purchase their products from cannabis dispensaries or other sources (e.g., online). By helping people self-select products based on individual symptoms and personal needs, this intervention offers a research template that can be applied to those using cannabis for chronic pain in various cannabis market structures (e.g., adult use or medical use only). Our findings demonstrate that an efficacy trial of this coaching intervention is appropriate for further study. We are currently recruiting for a large trial (*n* = 468) among Veterans with chronic pain in any state with legal adult-use cannabis (NCT06283862). Future research may consider tailoring the intervention approach to other populations with chronic pain or adapting intervention content into a training tool for cannabis dispensary staff and healthcare providers.

### Limitations and strengths

This study had several limitations. First, we had no control group and the changes observed from pre- to post-treatment could have been due to regression to the mean or other effects, including participant-therapist interactions. Second, our study population was small and consisted of predominantly male, older, White Veterans, some of whom had participated in our pilot qualitative study (Bergmans 2024), so our results may not generalize to other populations. Third, we did not thoroughly assess how coaching affected cannabis use patterns and whether specific products were associated with symptom changes. Fourth, we are missing feasibility and preliminary effectiveness data on 5 individuals, who may have had different perceptions and outcomes related to the intervention. These limitations are offset by study strengths, including that this study is the first such study to our knowledge that empirically assesses whether a coaching intervention could help reduce harm and maximize benefit from using legally available cannabis products for chronic pain. Importantly, our approach offered substantial flexibility over conventional clinical trials with cannabis-based study medications, in that participants could select their own products based on their specific constellation of symptoms and also use products that are available on the legal medical cannabis market.

## Conclusions

In this feasibility pilot study of coaching on cannabis use for chronic pain among Veterans, participants were satisfied with the intervention and reported improvements in pain symptoms. Our results support evaluating this intervention in a larger, efficacy trial.

## Data Availability

Data will be available from the authors upon reasonable request.
